# Supplementary material for: Taxonomic Study of Three Novel Paenibacillus Species with Cold-Adapted Plant Growth-Promoting Capacities Isolated from Root of Larix gmelinii
Source: Microorganisms. 2023 Jan 4;11(1):130. doi: 10.3390/microorganisms11010130 (PMC9867441; doi:10.3390/microorganisms11010130)
Supplement: Supplementary file 1 [file microorganisms-11-00130-s001.zip › microorganisms-2077786-supplementary.pdf]

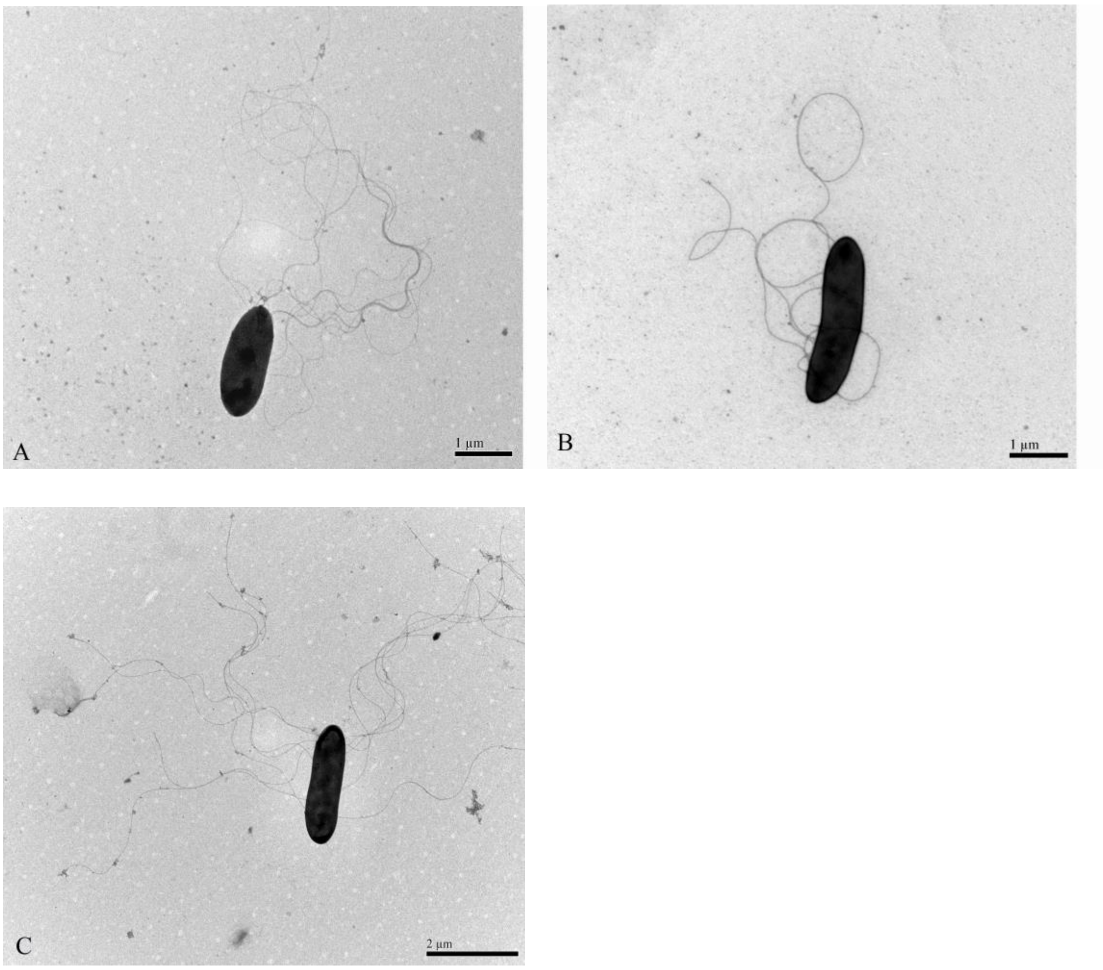

Figure S1 Transmission electron micrograph of cell of three isolates. A. *Paenibacillus endoradicis* sp. nov. T3-5-0-4 (Bar, 1µm); B. *Paenibacillus radicibacter* sp. nov N1-5-1-14 (Bar, 1µm); C. *Paenibacillus radicis* sp. nov. N5-1-1-5 (Bar, 2µm).

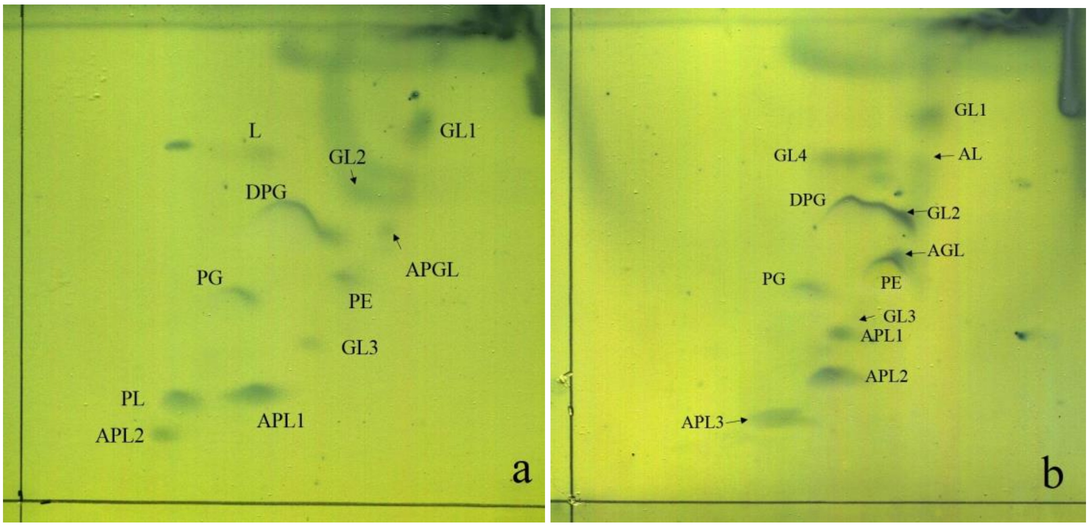

Han Xue, Yan Tu, Teng-fei Ma, Ning Jiang, Chun-gen Piao and Yong Li
Taxonomic study of three novel *Paenibacillus* species with cold-adapted plant growth-promoting capatities isolated from root of *Larix gmelinii*
Microorganisms

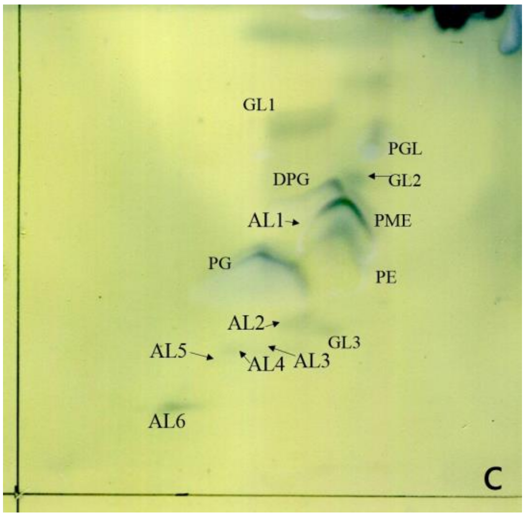

Figure S2. Polar lipid profiles of strains *Paenibacillus endoradicis* sp. nov. T3-5-0-4 (a), *Paenibacillus radicibacter* sp. nov N1-5-1-14 (b) and *Paenibacillus radicis* sp. nov. N5-1-1-5 (c). AGL, aminoglycolipid; APGL, aminophosphoglycolipid; APL, unidentified aminophospholipid; DPG, diphosphatidylglycerol; PE, phosphatidylethanolamine; PG, phosphatidylglycerol; PL, unidentified phospholipid; PME, phosphatidylmonomethylethanolamine; PGL, phosphoglycolipid; GL, unidentified glycolipid; L, unidentified Lipid; AL, unidentified aminolipid.

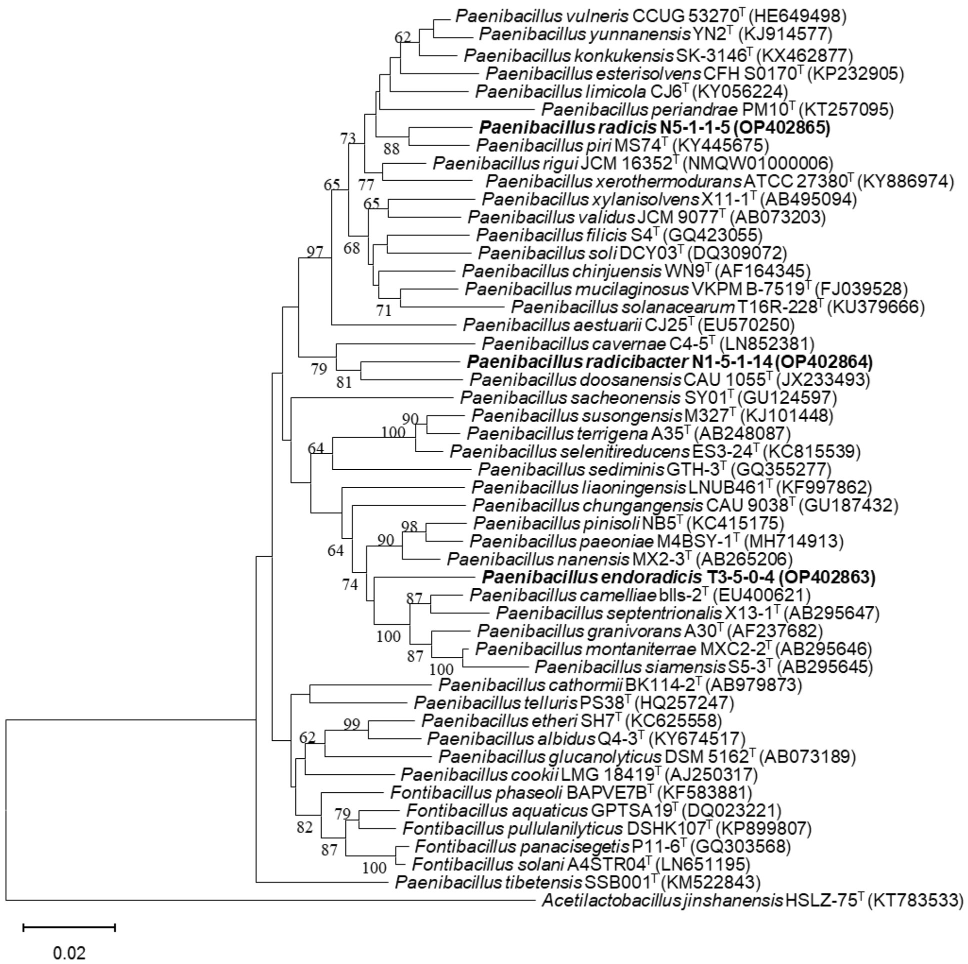

Figure S3. Neighbour-Joining tree tree illustrating the phylogenetic position of *Paenibacillus endoradicis* sp. nov. T3-5-0-4, *Paenibacillus radicibacter* sp. nov N1-5-1-14, *Paenibacillus radicis* sp. nov. N5-1-1-5 and other type species in the genus *Paenibacillus* based on 16S rRNA gene sequences. The sequence of *Acetilactobacillus jinshanensis* HSLZ-75^{T} was used as out-group. Bootstrap values (expressed as percentages of 1,000 replications) over 60% are shown at branching nodes. Bar, 0.02 substitutions per nucleotide position.

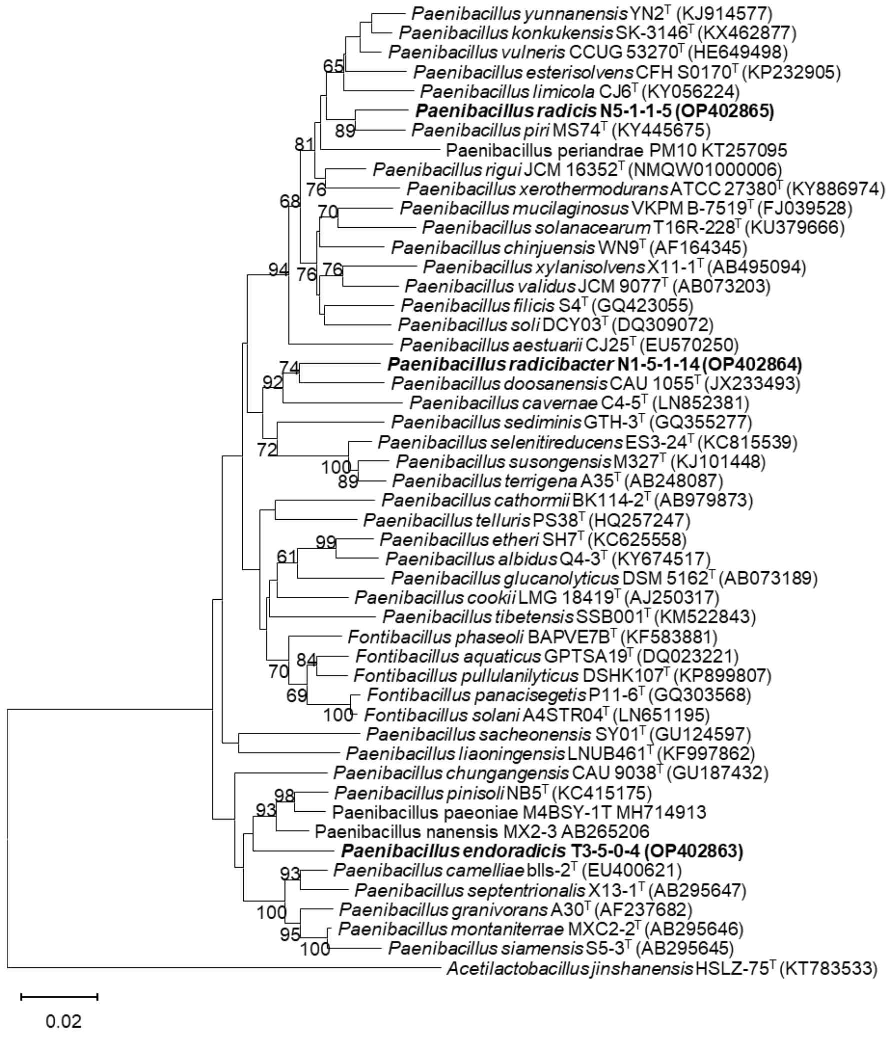

Figure S4. Minimum-Evolution tree illustrating the phylogenetic position of *Paenibacillus endoradicis* sp. nov. T3-5-0-4, *Paenibacillus radicibacter* sp. nov N1-5-1-14, *Paenibacillus radicis* sp. nov. N5-1-1-5 and other type species in the genus *Paenibacillus* based on 16S rRNA gene sequences. The sequence of *Acetilactobacillus jinshanensis* HSLZ-75T was used as out-group. Bootstrap values (expressed as percentages of 1,000 replications) over 60% are shown at branching nodes. Bar, 0.02 substitutions per nucleotide position.

Table S1. Carbon source utilization traits of three novel strains.

1. *Paenibacillus endoradicis* sp. nov. T3-5-0-4; 2. *Paenibacillus radicibacter* sp. nov N1-5-1-14; 3. *Paenibacillus radicis* sp. nov. N5-1-1-5. All of the three strains showed positive for starch, dextrin, D-maltose, D-trehalose, D-cellobiose, gentiobiose, D-turanose, β-methyl-D-glucoside, D-salicin, α-D-glucose and D-galactose, negative for N-acetyl-D-galactosamine, N-acetylneuraminic acid, 3-methyl glucose, D-fucose, D-arabitol, D-aspartic acid, D-serine, L-serine, glycyl-L-prolin, L-alanine, L-arginine, L-aspartic acid, L-glutamic acid, L-galactonic acid, mucic acid, quinic acid, D-saccharic acid, p-hydroxy-phenylacetic acid, D-lactic acid ethyl ester, citric acid, α-keto-glutaric acid, γ-amino-butryricAcid, α-hydroxy-

butyric acid, *β*-hydroxy-D,L-butyric acid, propionic acid and formic acid.

substrate	1	2	3
sucrose	+	-	+
stachyose	+	-	-
D-raffinose	+	-	+
α-D-lactose	+	-	+
D-melibiose	+	-	+
N-acetyl-D-glucosamine	-	+	-
N-acetyl-*β*-D-mannosamine	-	+	-
D-mannose	-	+	+
D-fructose	-	+	+
L-fucose	-	+	+
L-rhamnose	-	-	+
inosine	-	+	-
D-sorbitol	-	+	-
D-mannitol	+	+	-
myo-inositol	-	-	+
glycerol	+	-	+
D-glucose--6-PO_4	+	-	+
D-fructose-6-PO_4	+	-	+
L-histidine	-	+	-
pectin	+	-	+
D-galacturonic	-	-	+
L-galactonic	-	-	+
D-gluconic acid	+	+	-
glucuronamide	-	+	-
methyl pyruvate	-	-	+
L-lactic acid	-	-	+
D-malic acid	-	-	+
L-malic acid	-	-	+
bromo-succinic acid	-	-	+
tween 40	-	+	-
α-keto-butyric acid	+	-	-
acetoacetic acid	-	+	-
acetic acid	-	+	-

Table S2. Genes involved in motility and chemotaxis.

1. *Paenibacillus endoradicis* sp. nov. T3-5-0-4; 2. *Paenibacillus radicibacter* sp. nov N1-5-1-14; 3. *Paenibacillus radicis* sp. nov. N5-1-1-5.

Gene	Product	Pathway	Locus tag		
			1	2	3
cheA	two-component system, chemotaxis family, sensor kinase CheA	Chemotaxis	P15691_GM000612<br>P15691_GM004134<br>P15691_GM004164	P15694_GM001172	P15710_GM001334<br>P15710_GM003148<br>P15710_GM004620
cheB	two-component system, chemotaxis family, protein-glutamate methylesterase/glutaminase		P15691_GM000611<br>P15691_GM004138	P15694_GM001173	P15710_GM001335
cheC	chemotaxis protein CheC		P15691_GM000614	P15694_GM001170	P15710_GM001332
cheD	chemotaxis protein CheD		P15691_GM000615	P15694_GM001169	P15710_GM001331
cheR	chemotaxis protein methyltransferase CheR		P15691_GM004137<br>P15691_GM004163<br>P15691_GM004785	P15694_GM001293	P15710_GM005305<br>P15710_GM006978
cheW	purine-binding chemotaxis protein CheW		P15691_GM000613<br>P15691_GM003243<br>P15691_GM004135	P15694_GM001171	P15710_GM001333<br>P15710_GM002259<br>P15710_GM003147<br>P15710_GM007147
cheX	chemotaxis protein CheX		P15691_GM002349	P15694_GM004296	P15710_GM000209<br>P15710_GM001547
cheY	two-component system, chemotaxis family, chemotaxis protein CheY		P15691_GM000132<br>P15691_GM000602<br>P15691_GM002555	P15694_GM001182<br>P15694_GM002464	P15710_GM000256<br>P15710_GM000517<br>P15710_GM001344<br>P15710_GM004953<br>P15710_GM006435<br>P15710_GM006890<br>P15710_GM007926
motA	chemotaxis protein MotA		P15691_GM004926	P15694_GM004054	P15710_GM001220<br>P15710_GM003120

motB	chemotaxis protein MotB		P15691_GM004925	P15694_GM004055	P15710_GM001219<br>P15710_GM003119
mcp	methyl-accepting chemotaxis protein		P15691_GM000093<br>P15691_GM000094<br>P15691_GM001868<br>P15691_GM002025<br>P15691_GM002516<br>P15691_GM002517<br>P15691_GM003163<br>P15691_GM003964<br>P15691_GM004136<br>P15691_GM004560<br>P15691_GM004571	P15694_GM001441<br>P15694_GM001622<br>P15694_GM001667<br>P15694_GM001738<br>P15694_GM002055<br>P15694_GM002269<br>P15694_GM002755<br>P15694_GM003108<br>P15694_GM004042<br>P15694_GM004386	P15710_GM000911<br>P15710_GM001481<br>P15710_GM002061<br>P15710_GM002260<br>P15710_GM002909<br>P15710_GM003146<br>P15710_GM003573<br>P15710_GM004194<br>P15710_GM004932<br>P15710_GM006723<br>P15710_GM007120<br>P15710_GM007146<br>P15710_GM007432
fliA	RNA polymerase sigma factor for flagellar operon FliA	Flagella biosynthesis	P15691_GM000617	P15694_GM001167	P15710_GM001329
fliB	lysine-N-methylase		/	/	P15710_GM005665
fliC	flagellin		P15691_GM004899<br>P15691_GM005004	P15694_GM004100<br>P15694_GM004101	P15710_GM003051<br>P15710_GM003052
fliD	flagellar hook-associated protein 2		/	/	P15710_GM003074
fliE	flagellar hook-basal body complex protein FliE		P15691_GM000587	P15694_GM001197	P15710_GM001359
fliF	flagellar M-ring protein FliF		P15691_GM000588	P15694_GM001196	P15710_GM001358
fliG	flagellar motor switch protein FliG		P15691_GM000589	P15694_GM001195	P15710_GM001357
fliH	flagellar assembly protein FliH		P15691_GM000590	P15694_GM001194	P15710_GM001356
fliI	flagellum-specific ATP synthase		P15691_GM000591	P15694_GM001193	P15710_GM001355
fliJ	flagellar FliJ protein		P15691_GM000592	P15694_GM001192	P15710_GM001354
fliL	flagellar FliL protein		P15691_GM000599	P15694_GM001185	P15710_GM001347
fliM	flagellar motor switch protein FliM		P15691_GM000600	P15694_GM001184	P15710_GM001346
fliNY, fliN	flagellar motor switch protein FliN/FliY		P15691_GM000601	P15694_GM001183	P15710_GM001345
fliOZ, fliO	flagellar protein FliO/FliZ		P15691_GM000603	P15694_GM001181	P15710_GM001343

fliP	flagellar biosynthetic protein FliP		P15691_GM000604	P15694_GM001180	P15710_GM001342
fliQ	flagellar biosynthetic protein FliQ		P15691_GM000605	P15694_GM001179	P15710_GM001341
fliR	flagellar biosynthetic protein FliR		P15691_GM000606	P15694_GM001178	P15710_GM001340
fliS	flagellar protein FliS		P15691_GM005002	P15694_GM004097	P15710_GM003075
fliW	flagellar assembly factor FliW		P15691_GM004998	P15694_GM004103	P15710_GM003049
flgB	flagellar basal-body rod protein FlgB		P15691_GM000585	P15694_GM001199	P15710_GM001361
flgC	flagellar basal-body rod protein FlgC		P15691_GM000586	P15694_GM001198	P15710_GM001360
flgD	flagellar basal-body rod modification protein FlgD		P15691_GM000595	P15694_GM001189	P15710_GM001351
flgE	flagellar hook protein FlgE		P15691_GM000597	P15694_GM001187	P15710_GM001349
flgG	flagellar basal-body rod protein FlgG		P15691_GM004971<br>P15691_GM004972	P15694_GM004132<br>P15694_GM004133	P15710_GM003021<br>P15710_GM003022
flgK	flagellar hook-associated protein 1 FlgK		P15691_GM004996	P15694_GM004106	P15710_GM003046
flgL	flagellar hook-associated protein 3 FlgL		P15691_GM004997	P15694_GM004105	P15710_GM003047
flgM	negative regulator of flagellin synthesis FlgM		P15691_GM004994	P15694_GM004108	/
flhA	flagellar biosynthesis protein FlhA		/	P15694_GM001176	/
flhB	flagellar biosynthetic protein FlhB		/	P15694_GM001177	/
flhB2	flagellar biosynthesis protein		/	P15694_GM001210	/
flhF	flagellar biosynthesis protein FlhF		/	P15694_GM001175	/
flhG, fleN	flagellar biosynthesis protein FlhG		/	P15694_GM001174	/

Table S3. Genes involved in low temperature adaption.

1. *Paenibacillus endoradicis* sp. nov. T3-5-0-4; 2. *Paenibacillus radicibacter* sp. nov N1-5-1-14; 3. *Paenibacillus radicis* sp. nov. N5-1-1-5.

Gene	Product	Pathway	Locus tag		
			1	2	3
CspA	Cold shock protein	Transcription	P15691_GM002288<br>P15691_GM003570<br>P15691_GM004207<br>P15691_GM004338<br>P15691_GM005036	P15694_GM002387<br>P15694_GM002497<br>P15694_GM002580<br>P15694_GM002903	P15710_GM001119<br>P15710_GM001189<br>P15710_GM001654<br>P15710_GM003078<br>P15710_GM004548<br>P15710_GM004555

Han Xue, Yan Tu, Teng-fei Ma, Ning Jiang, Chun-gen Piao and Yong Li

Taxonomic study of three novel *Paenibacillus* species with cold-adapted plant growth-promoting capatities isolated from root of *Larix gmelinii*

Microorganisms

DnaK	Heat shock protein HSP70	chaperones	P15691_GM003656	P15694_GM004400<br>P15694_GM004794	P15710_GM002052
GrpE	Heat shock protein HSP70	chaperones	P15691_GM003655	P15694_GM004795	P15710_GM002053
RbfA	Ribosome-binding factor	Translation	P15691_GM003655	P15694_GM001147	P15710_GM001309
RNA helicase	Superfamily I DNA or RNA helicase	Replication, recombination and repair ;	P15691_GM002240<br>P15691_GM005073	P15694_GM001981<br>P15694_GM003229<br>P15694_GM004020	P15710_GM003151<br>P15710_GM004516
RNA helicase	Superfamily II DNA and RNA helicase	Replication, recombination and repair ;	P15691_GM002351<br>P15691_GM003577<br>P15691_GM003611<br>P15691_GM004341	P15694_GM002211<br>P15694_GM002917<br>P15694_GM004177<br>P15694_GM004293	P15710_GM000205<br>P15710_GM001677<br>P15710_GM004653<br>P15710_GM005659
RNA helicase	Superfamily II DNA or RNA helicase, SNF2 family	Transcription ; Replication, recombination and repair ;	P15691_GM002850<br>P15691_GM000214<br>P15691_GM002637<br>P15691_GM003465<br>P15691_GM004096	P15694_GM001605<br>P15694_GM002963<br>P15694_GM003593	P15710_GM004389<br>P15710_GM000910<br>P15710_GM000988<br>P15710_GM001754<br>P15710_GM006902<br>P15710_GM007011
gntR	DNA-binding transcriptional regulator	Transcription	P15691_GM001407<br>P15691_GM001565<br>P15691_GM001630<br>P15691_GM003997<br>P15691_GM004224<br>P15691_GM004254<br>P15691_GM004512<br>P15691_GM004617	P15694_GM000156<br>P15694_GM000160<br>P15694_GM001811<br>P15694_GM001961<br>P15694_GM002116<br>P15694_GM002202<br>P15694_GM003267<br>P15694_GM004549	P15710_GM000337<br>P15710_GM001213

Table S4. Genes involved in iron transport and siderophore production.

1. *Paenibacillus endoradicis* sp. nov. T3-5-0-4; 2. *Paenibacillus radicibacter* sp. nov N1-5-1-14; 3. *Paenibacillus radicis* sp. nov. N5-1-1-5.

Gene	Product	Pathway	Locus tag		
			1	2	3
afuA, fbpA	iron(III) transport system substrate-binding protein	Iron(III) transport	P15691_GM003866	P15694_GM000147<br>P15694_GM002526<br>P15694_GM003035<br>P15694_GM003409<br>P15694_GM003412	P15710_GM000547<br>P15710_GM002944<br>P15710_GM003222<br>P15710_GM003225<br>P15710_GM003360<br>P15710_GM003692<br>P15710_GM003849<br>P15710_GM003850<br>P15710_GM003851<br>P15710_GM003880<br>P15710_GM006188<br>P15710_GM006780<br>P15710_GM007337
afuB, fbpB	iron(III) transport system permease protein		P15691_GM003865	P15694_GM002089<br>P15694_GM002528<br>P15694_GM003033<br>P15694_GM003410	P15710_GM000546<br>P15710_GM002945<br>P15710_GM003223<br>P15710_GM003361<br>P15710_GM003690<br>P15710_GM003847<br>P15710_GM003879<br>P15710_GM006189<br>P15710_GM006781<br>P15710_GM007336
afuC, fbpC	iron(III) transport system ATP-binding protein		P15691_GM003867	P15694_GM002090<br>P15694_GM002527<br>P15694_GM003034	P15710_GM002946<br>P15710_GM003362<br>P15710_GM003691<br>P15710_GM006187<br>P15710_GM007335
efeB	deferrochelatase/peroxidase EfeB	Iron(II) transport	P15691_GM001992	P15694_GM004466	
efeO	iron uptake system component		P15691_GM001991	P15694_GM004467	

	EfeO				
ABC.FEV.A	iron complex transport system ATP-binding protein	Iron complex transport	P15691_GM000714<br>P15691_GM001174<br>P15691_GM001426<br>P15691_GM001661<br>P15691_GM001733<br>P15691_GM003217<br>P15691_GM003302<br>P15691_GM003704<br>P15691_GM003851	P15694_GM000577<br>P15694_GM001397<br>P15694_GM001834<br>P15694_GM002060<br>P15694_GM003395<br>P15694_GM003559<br>P15694_GM003610<br>P15694_GM003952<br>P15694_GM003990<br>P15694_GM004690	P15710_GM002208<br>P15710_GM002498<br>P15710_GM002566<br>P15710_GM003349<br>P15710_GM004512<br>P15710_GM005974<br>P15710_GM006551<br>P15710_GM006558<br>P15710_GM007527
ABC.FEV.P	iron complex transport system permease protein		P15691_GM000713<br>P15691_GM001175<br>P15691_GM001231<br>P15691_GM001232<br>P15691_GM001427<br>P15691_GM001428<br>P15691_GM001662<br>P15691_GM001735<br>P15691_GM001736<br>P15691_GM001899<br>P15691_GM001900<br>P15691_GM003214<br>P15691_GM003215<br>P15691_GM003301<br>P15691_GM003853<br>P15691_GM004368	P15694_GM000645<br>P15694_GM000646<br>P15694_GM001836<br>P15694_GM001837<br>P15694_GM002061<br>P15694_GM003396<br>P15694_GM003397<br>P15694_GM003560<br>P15694_GM003608<br>P15694_GM003609<br>P15694_GM003953<br>P15694_GM003988<br>P15694_GM003989<br>P15694_GM004691<br>P15694_GM004692	P15710_GM002209<br>P15710_GM002210<br>P15710_GM002567<br>P15710_GM002568<br>P15710_GM003320<br>P15710_GM003321<br>P15710_GM003584<br>P15710_GM003585<br>P15710_GM004513<br>P15710_GM004514<br>P15710_GM005646<br>P15710_GM005647<br>P15710_GM005972<br>P15710_GM005973<br>P15710_GM006552<br>P15710_GM006557<br>P15710_GM006740<br>P15710_GM006741<br>P15710_GM007526

ABC.FEV.S	iron complex transport system substrate-binding protein		P15691_GM000712<br>P15691_GM001173<br>P15691_GM001233<br>P15691_GM001425<br>P15691_GM001622<br>P15691_GM001663<br>P15691_GM001666<br>P15691_GM001734<br>P15691_GM001737<br>P15691_GM001902<br>P15691_GM003064<br>P15691_GM003855<br>P15691_GM004367<br>P15691_GM005038	P15694_GM000647<br>P15694_GM001835<br>P15694_GM001854<br>P15694_GM002062<br>P15694_GM003561<br>P15694_GM003607<br>P15694_GM003954<br>P15694_GM003987<br>P15694_GM004693	P15710_GM000604<br>P15710_GM002207<br>P15710_GM002570<br>P15710_GM003319<br>P15710_GM004099<br>P15710_GM004475<br>P15710_GM004477<br>P15710_GM004515<br>P15710_GM005397<br>P15710_GM005645<br>P15710_GM005971<br>P15710_GM006553<br>P15710_GM006556<br>P15710_GM006739<br>P15710_GM007525
mntH	manganese transport protein	Manganese/iron transport	P15691_GM004901	P15694_GM000609	P15710_GM005395<br>P15710_GM006083
entA	2,3-dihydro-2,3-dihydroxybenzoate dehydrogenase	Enterobactin production	/	P15694_GM001852	/
entB, dhbB, vibB, mxcF	bifunctional isochorismate lyase / aryl carrier protein		/	P15694_GM001849	/
entC	isochorismate synthase		/	P15694_GM001851	/
entE, dhbE, vibE, mxcE	2,3-dihydroxybenzoate-AMP ligase		/	P15694_GM001850	/

Table S5. Genes involved in phosphate solubilization and transport.

1. *Paenibacillus endoradicis* sp. nov. T3-5-0-4; 2. *Paenibacillus radicibacter* sp. nov N1-5-1-14; 3. *Paenibacillus radicis* sp. nov. N5-1-1-5.

Gene	Product	Pathway	Locus tag		
			1	2	3
aphD	aminoglycoside 2"-phosphotransferase	Organic phosphate solubilization	/	P15694_GM005023	/

Han Xue, Yan Tu, Teng-fei Ma, Ning Jiang, Chun-gen Piao and Yong Li
Taxonomic study of three novel *Paenibacillus* species with cold-adapted plant growth-promoting capatities isolated from root of *Larix gmelinii*
Microorganisms

phoA, phoB	alkaline phosphatase		/	P15694_GM004792	P15710_GM006716
phnA	protein PhnA	Degradation of phosphonates	P15691_GM003176	P15694_GM001873	/
phnB	PhnB protein		P15691_GM000918<br>P15691_GM001904<br>P15691_GM004621	P15694_GM000657<br>P15694_GM001591<br>P15694_GM003294<br>P15694_GM003832	P15710_GM000007<br>P15710_GM002613<br>P15710_GM002706<br>P15710_GM002793<br>P15710_GM006054<br>P15710_GM007937
phnC	phosphonate transport system ATP-binding protein		P15691_GM001166<br>P15691_GM003606	/	P15710_GM000776<br>P15710_GM002372<br>P15710_GM007860
phnD	phosphonate transport system substrate-binding protein		P15691_GM003605	/	P15710_GM000777<br>P15710_GM007637
phnE	phosphonate transport system permease protein		P15691_GM003607<br>P15691_GM003608	/	P15710_GM000774<br>P15710_GM000775<br>P15710_GM007858<br>P15710_GM007859
phnG	alpha-D-ribose 1-methylphosphonate 5-triphosphate synthase subunit PhnG		/	/	P15710_GM000784
phnH	alpha-D-ribose 1-methylphosphonate 5-triphosphate synthase subunit PhnH		/	/	P15710_GM000783
phnI	alpha-D-ribose 1-methylphosphonate 5-triphosphate synthase subunit PhnI		/	P15694_GM000144	P15710_GM000782
phnJ	alpha-D-ribose 1-methylphosphonate 5-phosphate C-P lyase		/	P15694_GM000143	P15710_GM000781
phnK	putative phosphonate transport system ATP-binding protein		/	P15694_GM000142	P15710_GM000780
phnL	alpha-D-ribose 1-methylphosphonate 5-triphosphate synthase subunit PhnL		/	P15694_GM000141	P15710_GM000778
phnM	alpha-D-ribose 1-methylphosphonate 5-triphosphate diphosphatase		P15691_GM002192	P15694_GM000140	P15710_GM000779
phnP	phosphoribosyl 1,2-cyclic phosphate phosphodiesterase		P15691_GM001202	P15694_GM002418	P15710_GM006417

phnW	2-aminoethylphosphonate-pyruvate transaminase		/	P15694_GM002087	/
phnX	phosphonoacetaldehyde hydrolase		/	P15694_GM002088	/
pstA	phosphate transport system permease protein	Phosphate transport	P15691_GM003477	P15694_GM002758	P15710_GM001484
pstB	phosphate transport system ATP-binding protein		P15691_GM003478	P15694_GM002757	P15710_GM001485
pstC	phosphate transport system permease protein		P15691_GM003476	P15694_GM002759	P15710_GM001483
pstS	phosphate transport system substrate-binding protein		P15691_GM003475	P15694_GM002761	P15710_GM001482<br>P15710_GM002218<br>P15710_GM005136

Table S6. Genes involved in phytohormone biosynthesis.

1. *Paenibacillus endoradicis* sp. nov. T3-5-0-4; 2. *Paenibacillus radicibacter* sp. nov N1-5-1-14; 3. *Paenibacillus radicis* sp. nov. N5-1-1-5.

Gene	Product	Pathway	Locus tag		
			1	2	3
trpA	tryptophan synthase alpha chain	L-tryptophan production; IAA production	P15691_GM004776	P15694_GM001283	P15710_GM001521<br>P15710_GM005316
trpB	tryptophan synthase beta chain		P15691_GM002916<br>P15691_GM004777	P15694_GM001284<br>P15694_GM001932	P15710_GM001520<br>P15710_GM004969<br>P15710_GM005315
trpC	indole-3-glycerol phosphate synthase		P15691_GM004779	P15694_GM001286	P15710_GM005313
trpD	anthranilate phosphoribosyltransferase		P15691_GM004780	P15694_GM001287	P15710_GM000010<br>P15710_GM005312
trpE	anthranilate synthase component I		P15691_GM004781	P15694_GM001288	P15710_GM005311
trpF	phosphoribosylanthranilate isomerase		P15691_GM004778	P15694_GM001285	P15710_GM005314
solA	N-methyl-L-tryptophan oxidase		/	/	P15710_GM000652<br>P15710_GM005126
aldB	aldehyde dehydrogenase	IAA production, IPA pathway	P15691_GM004230	P15694_GM001455	/
ALDH	aldehyde dehydrogenase (NAD+)		P15691_GM001715	P15694_GM003164	P15710_GM002662
amiE	amidase	IAA production, IAM pathway	P15691_GM001023<br>P15691_GM002228	P15694_GM001222	P15710_GM001000<br>P15710_GM006021

miaA	tRNA dimethylallyltransferase	CK biosynthesis and transformation		P15694_GM000614	P15710_GM002548
miaB	tRNA-2-methylthio-N6-dimethylallyladenosine synthase		P15691_GM000679	P15694_GM001109	P15710_GM001265

Table S7. Polar lipid profile of three novel strains and the type strains of the most closely related *Paenibacillus* species. strains: 1, *Paenibacillus endoradicis* sp. nov. T3-5-0-4; 2, *Paenibacillus paeoniae* M4BSY-1; 3, *Paenibacillus radicibacter* sp. nov N1-5-1-14; 4, *Paenibacillus doosanensis* CAU 1055^{T}; 5, *Paenibacillus radicis* sp. nov. N5-1-1-5^{T}; 6, *Paenibacillus rigui* WPCB173^{T}.

**1**	**2**[*]	**3**	**4**[#]	**5**	**6**[Δ]
DPG, PG, PE, APGL, GL1-3, APL1-2, PL, L	DPG, PG, PE, APL, PL	DPG, PE, AGL, PG, AL, GL1-4, APL1-3	DPG, PE, PG, LPG, APL	PME, PG, DPG, PE, PGL, GL1-3, AL1-6	PE, PG, PL1-3

DPG, diphosphatidylglycerol; PG, phosphatidylglycerol; PE, phosphatidylethanolamine; APGL, aminophosphoglycolipid; GL, unidentified glycolipid; APL, unidentified aminophospholipid; PL, unidentified phospholipid; L, unidentified Lipid; AGL, aminoglycolipid; AL, unidentified aminolipid; LPG, lysyl-phosphatidylglycerol; PME, phosphatidylmonomethylethanolamine; PGL, phosphoglycolipid.
Data taken from: *, Yan & Tuo (2018); #, Kim et al. (2014); Δ, Baik et al. (2011).

Han Xue, Yan Tu, Teng-fei Ma, Ning Jiang, Chun-gen Piao and Yong Li
Taxonomic study of three novel *Paenibacillus* species with cold-adapted plant growth-promoting capatities isolated from root of *Larix gmelinii*
Microorganisms
